# The Virtual Environment for Rapid Prototyping of the Intelligent Environment

**DOI:** 10.3390/s17112562

**Published:** 2017-11-07

**Authors:** Yannick Francillette, Eric Boucher, Abdenour Bouzouane, Sébastien Gaboury

**Affiliations:** Université du Québec à Chicoutimi, LIARA, Chicoutimi, QC G7H 2B1, Canada; eric.boucher1@uqac.ca (E.B.); abdenour_bouzouane@uqac.ca (A.B.); sebastien_gaboury@uqac.ca (S.G.)

**Keywords:** smart home, intelligent environment, simulation, sensor, visualisation

## Abstract

Advances in domains such as sensor networks and electronic and ambient intelligence have allowed us to create intelligent environments (IEs). However, research in IE is being held back by the fact that researchers face major difficulties, such as a lack of resources for their experiments. Indeed, they cannot easily build IEs to evaluate their approaches. This is mainly because of economic and logistical issues. In this paper, we propose a simulator to build virtual IEs. Simulators are a good alternative to physical IEs because they are inexpensive, and experiments can be conducted easily. Our simulator is open source and it provides users with a set of virtual sensors that simulates the behavior of real sensors. This simulator gives the user the capacity to build their own environment, providing a model to edit inhabitants’ behavior and an interactive mode. In this mode, the user can directly act upon IE objects. This simulator gathers data generated by the interactions in order to produce datasets. These datasets can be used by scientists to evaluate several approaches in IEs.

## 1. Introduction

Intelligent environments represent a major evolution in human societies. These systems are in line with Mark Weiser’s vision of computing [1,2,3]. They are distributed in the environment and continually interact with several people at the same time. Weiser and scientific community call this vision “ubiquitous computing”. This is in contrast with the vision of computing where a computer is set in a room and is used only by a few skilled users.

This kind of system can have a great impact on life and human society [4]. In fact, we can add intelligence to several kinds of environments, and the nature of the environment conditions the nature of this impact. For example, we can add these systems to housing and build “smart homes ”. Smart homes can control electronic devices in rooms in order to provide occupants with guidance and assistance so as to improve comfort and energy consumption. They can manage heating, lighting, and electric cookers to reduce electricity consumption. These systems are particularly interesting in the healthcare domain because they can help frail and disabled people recover a degree of autonomy. At a higher level, intelligent environments can help societies to manage resources. For example, smart homes can reduce the need for caregivers at home [5]. Smart cities can manage the city’s assets. In a general way, an intelligent environment (IE) provides users with information, automation and an interaction medium in order to be more efficient.

The concept of IEs creates opportunities, but also raises several issues. Efficiency, robustness, and quality of decision are examples of issues in this domain. Indeed, because IEs are systems with close interactions and direct impacts on the real world, it is important to address these issues in order to increase confidence and promote the use of these systems. Also, cost reduction is necessary in order to implement systems in several locations.

Removing technical and economic obstacles involves experimentation with new approaches and specific designs. These experiments require infrastructure, or at least datasets. Unfortunately, access to intelligent environments or suitable datasets is not easy. Some datasets are available for researchers in the activity recognition domain. These datasets come from previous experiences. Usually, they contain data generated from sensors, a map of the environment, and experiment scenarios. WSU CASAS (Washington State University Center for Advanced Studies in Adaptive Systems) datasets are examples of these datasets [6]. However, these datasets may come from environments, sensors, and scenarios that do not fit with new experimental purposes. Indeed, in order to be useful in the study of a new approach, a dataset must be generated with particular sensors. These sensors have to be in a particular location and configuration and they have to be used in a defined way. For example, a radio-frequency identification (RFID) based technic will require that RFID antennas are in particular locations with an appropriated orientation. Unfortunately, it is not easy or possible for a user to build his/her own IE to generate an appropriate dataset. Indeed, an IE can be very expensive because of sensor, device and building costs. Moreover, because the data come from human activities and experiments have to be conducted for extended periods of time, the process of building datasets is time-consuming. Finally, some experiments can be difficult to conduct because of a lack of suitable participants, for example with respect to people with a particular handicap.

Simulation software is a tool that can help IE designers and researchers to overcome these problems [7]. Indeed, a simulation tool allows users to save money because it does not require: (1) the purchase of physical devices; or (2) a physical room. Moreover, these tools make performing experiments easier because: (1) candidate behavior can be simulated with artificial intelligence (AI), so physical candidates are not mandatory; (2) due to the the previous reasons, experiments can be performed as many times as necessary; and (3) users can adapt IE settings such as types, positions, and number of sensors quickly.

In this paper, we focus on smart home simulation. As presented before, smart homes form a category of IE. In this category, intelligence is added to residences. Smart homes can enhance comfort at home and help frail and disable people to recover autonomy. We can find several smart home simulator propositions in the literature [8,9,10,11,12,13,14,15,16,17,18]. These solutions help researchers to generate datasets to study issues and new approaches in the smart home domain. However, it is important to highlight the fact that simulator features affect the kinds of studies that can be conducted. For example, the nature of sensors and actuators affects experiment possibilities. If a simulator provides users with only contact sensors, researchers cannot study several approaches for activity detection. The consequences are the same if the granularity level of data is too high. Inversely, when a simulator generates the same kind of data as physical sensors, it allows users to study the same approaches as with physical sensors. For example, if it generates radio-frequency identification (RFID) signal strength, researchers can study algorithms based on these devices. If it directly provides an “*x*, *y*, *z*” vector, researchers cannot use the same approaches.

In addition to an appropriated simulation approach, a simulation tool must provide users with several features to be as helpful as possible. Firstly, it should be flexible and allow users to add new devices. Secondly, it should provide a simple and intuitive way to design the environment. However, the design approach must allow the creation of a wide range of IEs. Finally, it must support the creation of several kinds of scenarios. As for designing the environment, the approach to instantiating the scenario must be simple and intuitive.

In this paper, we present our solution for smart home simulation. This solution addresses the points we have introduced before. Moreover, it is open source and aims to propose a simple way to design smart homes and scenarios.

This paper is structured as follows. The next section introduces the background related to our research. It defines the concepts of smart homes, sensors, and simulation. Section 3 presents the related works. Section 4 introduces the architecture of our simulator and its implementation. Section 5 presents the generation of a dataset with our simulator. Section 6 presents a case study using our approach. Finally, Section 7 presents conclusion and future works.

## 2. Background

Before exploring IE simulation, it is important to define the main concepts. In this section, we define the concepts of smart homes, sensors, simulations, and virtual smart homes.

### 2.1. Smart Homes

**Figure 1:**
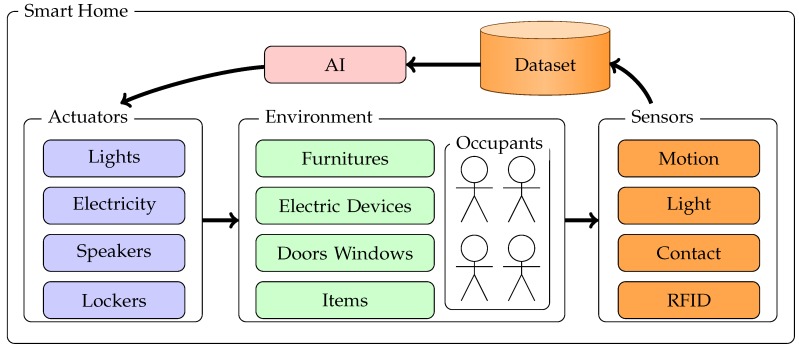
The main components of an intelligent environment (IE). AI: artificial intelligence; RFID: radio-frequency identification.

As mentioned before, smart homes form a category of IEs. In fact, the smart home is an application of the IE principle in the home domain. Moreover, it can also be seen as an extension of home automation. Indeed, home automation aims to increase comfort at home by automating tasks and making some of them easier. For example, some devices will clean floors, lock or unlock doors or windows, and activate fans or lights with few actions from the inhabitants. In a general way, these devices will provide users with a remote interface to control or perform settings. This point is the main difference between home automation and the smart home domain. Indeed, home automation generally requires inhabitants’ decisions, whereas smart homes involve an form of artificial intelligence (AI) that performs some decisions [19,20]. Basically, this AI is designed to reach some objectives. It collects data from sensors, computes if the environment needs adjustments to reach objectives, and uses actuators to adapt the environment. Figure 1 shows a scheme of the main smart home components.

We note that the smart home is a type of interactive system. Indeed, there is an interaction loop between the inhabitants and the smart home. Inhabitants (and external entities) act on elements of their environment. These actions affect the sensors. The AI interprets data generated by sensors, and then acts on some elements of this environment in response. The goal is to maintain the environment in a state that satisfies the AI objectives.

### 2.2. Sensors and Actuators

Sensors and actuators play a crucial role in the system. Indeed, they are sensitive organs and actuating drivers, respectively. Basically, a sensor is a device which acquires a physical quantity and converts it into usable output. The element that performs this conversion is called a transducer. This simply converts one form of energy to another. In the case of sensors, the transducer converts a physical quantity into electrical (or analogical) output. In the case of actuators, the transducer converts an electrical input into physical output.

In pervasive systems, several elements affect the choice of sensors and actuators [5]. The first type of element is related to environmental factors. For example, we can cite the size of the environment, temperature, and humidity, etc. The second type of element is related to the sensor’s characteristics: sensitivity, error rate, and range for instance. The last type of element is related to economic factors such as cost, lifetime, or availability.

### 2.3. Simulation and Dataset Generation

Simulation tools are helpful in the investigation of particular systems. Indeed, they allow designers and researchers to study outcomes of an event on a system without requiring an experiment on real systems. Consequently, they provide a solution to the economic and logistic issues. Basically, a simulation is an imitation of behaviors of a real-world system or process. However, in a general way simulations do not reproduce the exact behavior of all parts of the system they mimic. They focus on key elements and make some abstractions. Their overall goal is to produce the same effects as the real system.

In the smart home domain, simulations aim to reproduce behaviors of sensors, actuators, furniture, inhabitants, and electronic devices. This allows users to experiment implementations, study the impacts of interactions on entities, and generate datasets. In the case of dataset generation, the main objective is not to generate the same dataset exactly as a real system would with the same scenario. It is more important that generated datasets reflect events occurring in an environment, and these events must follow the same patterns, whether they are in the real world or simulated. In other words, it is not mandatory that the simulation model generate exactly the same data that real sensors produce. However, it is necessary that the data reproduce the same patterns. Consequently, simulation users can study real patterns from simulated data.

According to Synnott et al. [7], there exist two main approaches for smart home simulation: model-based and interactive approaches. Model-based approaches use activity models. These models define the order, time taken, and probability of events occurring during the accomplishment of specific activities. These approaches can be used to define activities over long periods of time and generate large datasets. However, according to Synnott et al. [7], it can be difficult to intuitively and accurately act on models to generate significant differences in activity completion.

Interactive approaches propose platforms to interact with sensors. In contrast to model-based approaches, these approaches focus on the modeling environment and environmental components rather than activities.

## 3. Related Works

We can find some works in the literature to generate datasets or simulate smart homes. In this section, we review these propositions according to analysis criteria introduced in the next subsection. Our selection criteria for the analysis are the following. We have focused on works published since 2012 that appear in response to the keywords: “smart home simulation” and “intelligent environment simulation”.

### 3.1. Analysis Criteria

The following analysis criteria are considered for the review:

- What is the approach? This question aims to identify the approach used for the simulation.
- What is the collection of sensors and actuators? This question aims to identify a list of sensors and actuators integrated into the simulator.
- Does it support multi-occupants? The answer to this question indicates whether the proposition supports multi-occupants.
- Does the proposition support environment events? The answer to this question indicates if the solution allows users to define environment events (i.e., events that are not related to activity completion such as a device failure). We define three answers:
  - No: there is no way to define environment events.
  - Engine extension: the propostion does not provide the users with a dedicated method. However, it is possible to extend (because the proposition is open-source for example) the engine to add this feature.
  - Yes: the proposition provides users with a dedicated method.
- Does the proposition support actuator control? The answer to this question indicates if the solution allows users to control actuators. Possible answers are the same as the previous question.

### 3.2. Presentation

SIMACT is an open-source smart home simulator proposed in [15]. The authors use the model-based approach to design their simulator. SIMACT provides an interface to design the smart home and scripts. In the scripts, the user has to define the sequence of steps involved in the realization of activities. The user has to define parameters such as the completion time of a step and the objects that are involved. The simulator uses Java and runs into a 3D environment designed with SketchUp. The simulator stores data about object interactions in a database. In fact, SIMACT proposes only contact sensors; it detects when an action is performed on an object and stores the contact information.

PerSim 3D is an enhancement of PerSim 1.5 proposed in [13]. The two versions use a model-based approach. However, PerSim 3D proposes a virtual model for the sensor behavior. The authors try to model virtual behavior that is similar to real behavior. However, this is very difficult because of the simulation model. PerSim provides users with several categories of sensors. It also allows the user to design the architecture of the home. Like SIMACT, the user can play and see the simulation through a 3D rendering of the scene. PerSim provides the user with more kinds of sensors than SIMACT, however, PerSim seems not to record the simulated raw data of sensors. In fact, sensor data is transformed in order to determine whether it is activated or not.

UbikSim is a simulator of the intelligent environment proposed in [21,22]. The authors use the model-based approach to design this simulator. UbikSim uses multi-agent based simulation (MABS) to perform the occupants’ behaviors. More precisely, it uses the Java library Multi-agent Simulator of Neighborhoods (MASON) [23] for the simulation of occupants. Consequently, UbikSim supports simulations involving several occupants. It uses Sweet Home 3D [24] (a computer-aided design software for designing interiors) to support the design of the environments. UbiKSim provides users with two binary sensors, door sensors, and pressure sensors. It works in a 3D world and provides a real-time rendering. UbikSim requires that users program in Java in order to design experiments.

In [12], the authors propose a simulator which can generate data from simulated sensors. They use a model-based approach. The authors split their sensors in two categories: motion and non-motion. The non-motion sensors are like an environmental variable. They are increased while the inhabitant performs an action where they are involved. For example, water consumption is increased when the inhabitant uses a faucet. The motion sensors are sensors that detect if the inhabitant is in a particular zone. Then, it computes a distance in order to simulate a signal strength, for example. This simulator works in a 2D world and it does not provide a real-time rendering.

The Intelligent Environment Simulation (IE Sim) [10,11] is used to generate simulated datasets that capture normal and abnormal activities of daily living (ADLs) of inhabitants. This tool provides users with a 2D graphical top view of the floor plan to design a smart home. It proposes different types of sensors such as temperature sensors, pressure sensors, etc. Simulation is also performed in a 2D world. However, in contrast to the previous solution, this proposition uses the interactive approach to generate datasets.

In order to generate data from the interactions of the inhabitants, Ariani et al. [14] propose a smart home simulation tool that uses ambient sensors. The solution provides users with an editor that allows the design a floor plan for a smart home by drawing shapes on a 2D canvas. Once this step is over, we can add ambient sensors to the virtual home. The solution can simulate binary motion detectors and binary pressure sensors.

In [9], Park et al. propose a simulator to generate inhabitants’ datasets for classification problems. Their simulator is built with Unity3D [25] and proposes a 3D environment. They use an interactive approach to generate datasets. They have analyzed collected data to generate a user activity reasoning model in a virtual living space.

OpenSHS is a recent proposition to generate datasets [8]. It is a hybrid, open-source cross-platform which is in 3D thanks to the use of Blender [26] and Python. They use a hybrid approach to generate datasets. During simulations, users control an avatar. However, the solution proposes a fast-forward mechanism to allow users to mimic, but not perform a whole activity in real time. This mechanism uses a replication algorithm to extend and expand the dataset. It simply copies and repeats the existing state of all sensors and devices during the specified period.

### 3.3. Discussion

Table 1 summarizes the different works. This table highlights several trends. If we focus on the first criterion, we note that model-based and interactive approaches are used. In fact, model-based approaches tend to sacrifice the granularity of interaction, but allow users to generate large datasets quickly. On the other hand, interactive approaches tend to sacrifice generation speed, but propose smaller granularity. In fact, interactive approaches tend to consume the same time as in real-world experimentation. However, we can note that OpenSHS [8] and IE Sim [10] propose hybrid approaches to increase the realism of the simulated data.

Now let us focus on the second criterion. We can see that binary sensors and binary data are widely implemented. Indeed, all solutions propose at least this kind of sensor. In fact, we can suppose that this is because this type of sensor is relatively frequent in studies. Moreover, the kind of binary sensors present in the state of the art is quite simple to simulate. Indeed, generally they detect when an entity enters in a particular zone or when the occupant triggers a particular action (for example the “open” action). Kormanyos et al. [12] provide the only solution that includes numeric sensors (water and electricity consumption, temperature, and RFID). These sensors are more complicated to simulate because they require modeling of more complex physical phenomenon. However, these kinds of sensors can be used in several approaches for activity recognition. For example, Halle et al. [27] propose an approach based on electricity signature of electric devices.

If we focus on the third criterion, we can note that only three propositions support multiple occupants. However, OpenSHS proposes a partial support of this feature. In fact, this simulator needs to first record the actions of the occupant and it is able to play these actions in the background while another occupant is acting.

We can note that there is no solution that proposes dedicated methods to implement actuators into the environment. Consequently, no solution proposes tools to allow AI to manage the environment. We can explain this by the fact that these propositions focus on dataset generation or visualization. We can also see that there is no solution that can allow users to define environmental events. Due to the fact that OpenSHS, UbikSim and SIMACT are open-source, it is possible to extend these engines to add these features. Indeed, users can define their own sensors/devices with a particular behavior. However, that means that the user writes a new version of the engine for each new scenario. This solution has several drawbacks because scenarios are linked to core elements of the engine. That can limit evolution perspectives.

In conclusion, we can see that there is no solution that proposes several categories of sensors, control of actuators, and performs environment event and multiple-occupant support. In this paper, we propose a solution that involves numeric and binary sensors, control of actuators, and support for environmental events and multiple occupants. This solution is implemented with Unity3D and is open source.

## 4. Proposition

In this section, we introduce our IE simulator. Firstly, we present an overview of the general model. Then, we describe each concept used to define our simulator. We conclude this section by introducing the implementation.

### 4.1. Overview of the Proposition

**Figure 2:**
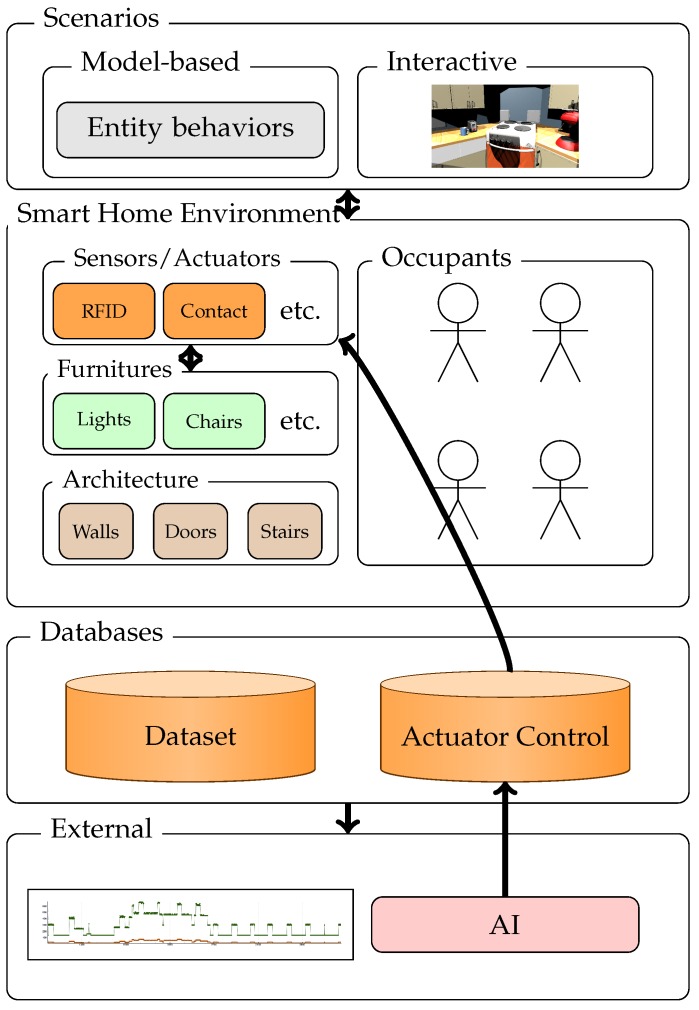
Overview of our proposition.

Figure 2 shows the architecture of our simulator. It is split in four parts. We named the first one the “Smart Home Environment” and it is related to all core components that perform simulations. It refers to our models for sensors, physics, resolution of interactions, etc. The second one is named “Scenarios” and contains elements that allow users to run different scenarios. The third one refers to the databases used in the simulation. The last one alludes to external elements that can interact with the simulator.

### 4.2. Smart Home Environment

We can classify elements of the smart home environment into four categories:

1. “Architecture” refers to position of walls, floors, stairs, etc.
2. “Furniture” refers to all objects and devices for daily life. For example, drawers, beds, Toilets, tables, chairs, plates, doors, etc.
3. “Sensors and actuators” refer to all devices added to transform the environment into an intelligent environment.
4. “Occupants” refer to all living beings that will interact with the environment. In our proposition we mainly refer to humans, but this can be extended to pets, for example.

During a scenario, occupants interact with elements of the “furniture”, “sensors” and “occupants” groups. In order to detect usable elements around an occupant, we draw a sphere box around him and we consider as potential interactive elements all elements that collide with this sphere box. We adopt an approach used in “ray casting problems” [28] to detect potential obstacles between the occupant and the entity. This consists of drawing a ray from one entity to another to detect all elements between them. This procedure allows our simulation engine to prevent occupants from acting on objects through walls or doors.

In the next subsections, we will introduce our simulation models for sensors and actuators. For each sensor, we will resume the sensor principle, and then our simulation model for this sensor. Table 2 resumes our approaches for sensors.

#### 4.2.1. RFID

RFID antennas and RFID tags are used in literature to track objects’ locations and deduce occupants’ activities [29,30]. The principle is quite simple. One or several RFID tags are put on objects and several RFID antennas are set in the environment. RFID antennas send signals that are caught by RFID tags in range. RFID tags activate when they receive a signal and answer to the antenna which computes signal strength between it and the tag. Signal strength decreases according to the distance between the two entities. Approaches use signal strength to deduce the objects’ locations.

We draw a detection zone (this detection zone can be a half sphere, a cone, etc.) for each RFID antenna. An RFID tag is a special object which belongs to a specific collision group. Each time an RFID tag collides with a detection zone, a Raycast is sent from the antenna to the tag to detect obstacles. If there is no obstacle between the two entities, we apply the following formula to compute the received signal strength indication (RSSI):

(1) RSSI(x)=(−9.1333ln(x)−10.726)×(1+cos(a))+noise_function()

where *x* and *a* are the distance and the angle between the antenna and the RFID tag, respectively. noise_function() is a function to generate noise and *s* is the seed of this function.

Basically (−9.1333ln(x)−10.726) [30] is a part of the formula that computes signal strength for an RFID tag that is in front of an RFID antenna (the angle between the antenna and the tag is equal to 90 degrees). However, signal strength decreases according to the angle. We apply the formula (1+cos(a)) to decrease signal strength. We use the function noise_function() to generate noise and add variations.

Our goal is not to build a simulation that generates the exact same signal strength as a real antenna. In fact, this objective is hard to reach because too many variables affect signal strength in the real world. Our objective is to reproduce the following key aspect of signal strength:

1. Signal strength is stronger if the tag is in front of the antenna (the angle between the antenna and the tag is equal to 90 degrees). The signal strength is decreased according to the distance between the tag and the antenna. This aspect is covered by the function (−9.1333ln(x)−10.726) [30].
2. Signal strength is degraded according to the angle between the antenna and the tag. This aspect is covered by the function (1+cos(a)).
3. Signal strength is affected by noise and the signal strength is not necessarily the same even with a tag that does not move. This aspect is covered by noise_function().

**Figure 3:**
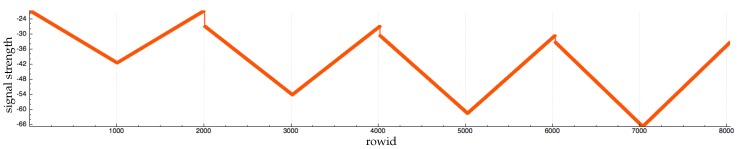
Evolution of RFID signal strength. In rows [0, 2000], the tag is approximately 1 m away from the antenna. In rows [2000–4000], the tag is approximately 4 m away from the antenna. In rows [4000–6000], the tag is approximately 10 m away from the antenna. In rows [6000–8000], the tag is approximately 10 m away from the antenna.

**Figure 4:**
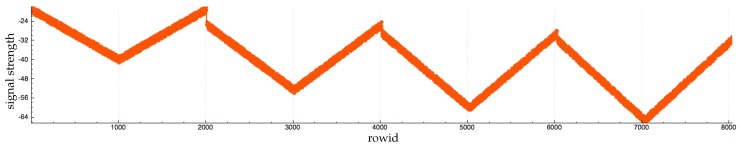
Evolution of RFID signal strength. In rows [0–2000], the tag is approximately 1 m away from the antenna. In rows [2000–4000], the tag is approximately 4 m away from the antenna. In rows [4000–6000], the tag is approximately 10 m away from the antenna. In rows [6000–8000], the tag is approximately 10 m away from the antenna.

**Figure 5:**
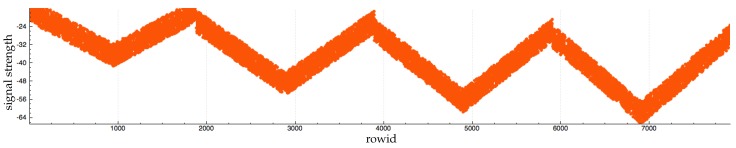
Evolution of RFID signal strength. In rows [0–2000], the tag is approximately 1 m away from the antenna. In rows [2000–4000], the tag is approximately 4 m away from the antenna. In rows [4000–6000], the tag is approximately 10 m away from the antenna. In rows [6000–8000], the tag is approximately 10 m away from the antenna.

Figure 3, Figure 4 and Figure 5 show RFID data generated by a tag moving around an antenna. Figure 3 shows the dataset without noise. This kind of data does not exist in the real world because it is almost impossible to work in an environment without interference. However, we can see that the first two aspects of signal strength are covered. Figure 4 shows the same dataset generation with our noise function that adds to the RSSI a number randomly chosen in the interval [0, 3]. Figure 5 shows the same dataset with our noise function that adds a random number between 0 and 10 to RSSI. In this generation, we keep the tag fewer seconds at each location. The tag is kept at the same height and there is no obstacle between the tag and the antenna.

We note that Figure 5 contains fewer rows than Figure 3 and Figure 4. This is because at times the antenna lost contact with the tag in Figure 5.

If there is an obstacle between the RFID tag and the antenna, we alter the signal strength according to a degradation function linked to the obstacle. Basically, this allows us to simulate the fact that some tags can be detected through walls or doors, but with a poor signal strength.

#### 4.2.2. Pressure Plate

Pressure plates are devices that activate (and can return pressure) when entities are on them. They can be used to detect an entity location or occupant falls. The principle is quite simple; when an entity is on a pressure plate, the pressure it exerts triggers the sensor.

In order to simulate this behavior, firstly all entities of the environment have a mass. When an entity collides with the collision box of a pressure plate, it reads the physical information of entities to compute the pressure. We link our pressure plate with a threshold and if the pressure is higher than the threshold the sensor activates.

#### 4.2.3. PIR Motion

Passive infrared (PIR) movement detectors react when an entity moves into their detection range. We simulate these kinds of sensors by using a collision box. Basically, when an entity collides with the sensor’s collider box, we record the entity’s position. The sensor is activated if this position changes. Moreover, in our simulator, after the activation, the sensor is deactivated and it is enabled to activate for a configurable period of time.

#### 4.2.4. Contact Sensor and Flow Meter

Contact sensors and flow meters are binary sensors that can be used on doors or drawers to deduce their state (opened or closed) [31]. In our simulator, we catch the action sent on the item to change the state of the sensor. For example, if an inhabitant sends an action open on a door, the sensor detects the action as open, and then it changes its state from “*true*” to “*false*”.

#### 4.2.5. Ultrasound and Infrared Sensors

IR (Infrared) and ultrasound sensors send signals and report the distance where the signal is broken. We use the Raycast concept to simulate this behavior. When Raycast collides with an entity, we record the distance between this entity and the sensor. We allow the range of detection of these sensors to be changed.

#### 4.2.6. Electricity

Variation in electricity consumption can be used in the activity recognition domain [27,32]. Our simulator is able to generate data on environment electricity consumption. Electricity in our simulator uses the following model. It is provided by *n* phases. A phase has active and reactive power. When an electronic device has changes in its electricity consumption, it sends an action to the electricity manager. The action contains data about change in each phase. The electricity manager records the state of each phase *x* times per second. A script is linked to each device. This script specifies the electrical behavior of the device. It has to be defined by the user of the simulator. We use the behavior tree formalism [33] as models for these scripts. This formalism is described in the next subsection.

### 4.3. Scenario

Our simulator provides users with two modes (interactive and model-based) to generate datasets. In the interactive mode, users directly control an avatar and interact with the virtual environment. In the model-based mode, scripts are attached to occupants and they perform activities defined by these scripts. We use the same formalism (behavior tree) as for electricity for these scripts.

Behavior trees are a formalism used in planning and the video game community [34,35,36]. They are used frequently to implement the AI of non-playable characters (NPCs) in games. One of the main motivations for this paradigm is the use of a model that is easy to understand graphically and efficient to execute.

Basically, a behavior tree is a directed rooted tree that hierarchizes the executions of tasks that are performed by an entity. In this tree, leaf nodes represent an atomic task that can be directly executed, such as control of the state of an entity or movement to a position. Intermediate nodes represent activities undertaken by their children. They control how their child nodes evolve. We can find several names for this kind of node in the literature (control flow node, composite) [37]. In this paper, we use the name “composite node”. We more precisely describe composite nodes below.

When the behavior tree is executed, nodes can be in one of the following states:

- **Not running**: the node has never been started.
- **Running**: the node has been started but is not finished yet.
- **Succeeded**: the node is finished with success.
- **Failed**: the node finished with failure.

It is important to notice that in this model, failure does not mean an error. The state “failed” means that the action has not been done and the action cannot be done in the future. For example, if the task consists of completing a task before a certain time, the state “failed” means the time is over and the task has not been completed. The state “succeeded” means that the action has been done. Basically, the succeeded and failed states represent two end states for the node. The parent of a node will act according to the end states of their children and their nature.

The execution of a behavior tree is based on discrete updates. An update performs a single depth-first traversal of the tree recursively from the root. In this traversal, each node computes its state. If the node is a composite node, it defines how its children are traversed. It is important to notice that in behavior trees, the order of children has a meaning. Children are usually aligned horizontally and the first node is the leftmost. Most of the time, children are traversed from left to right until a child reaches a particular state.

Several composite nodes with particular traversal logic can be created according to the domains and the needs of behavior trees users. However, the three following types of composite logic are widely implemented. Table 3 resumes these composite logic states:

- **Sequence**: It executes node sequentially. It starts from the first one and each time a node ends in a succeeded state it starts the next one. If a child ends in failed state, it ends in a failed state.
- **Selector**: It executes nodes sequentially until one ends in a successful state. In this case, it ends in a succeeded state. If all children are in a failed state, it ends in a failed state.
- **Decorator**: It transforms the end state (succeeded or failed) of its only child. As a decorator we can present:
  - **Repeat**: It repeats the processing of the child. It can repeat until a number of succeeded states is reached.
  - **Inverter**: It inverts the ending state of its child (failed is transformed to succeeded for example).

One advantage of the behavior tree is the ability to compose complex behaviors from a set of simple elements. Additionally, it allows a smart home designer to define the behavior of an entity without worrying about how simple tasks work. In fact, he/she just has to focus on the sequencing of tasks. Moreover, it allows them to reuse old behaviors as nodes for new ones. In our approach, we use the name “behavior components” for leaf nodes. A behavior component is a script that defines an objective and manages the entity in order to reach it. Moreover, it can have several parameters that allow users to create variations among the same scenario.

Let us use a design scenario to show the use of behavior trees for smart home design. Firstly, we consider the following behavior components:

- **Go to**: The inhabitant goes to a location.
- **Take**: The inhabitant takes an item. The item must be near the inhabitant otherwise this component ends with a failure.
- **Release**: The inhabitant releases an item. The inhabitant must have obtained this item, otherwise this component ends with a failure.
- **Activate/Deactivate**: The inhabitant (de)activates a device. The device must be near the inhabitant, otherwise this component ends with a failure.

**Figure 6:**
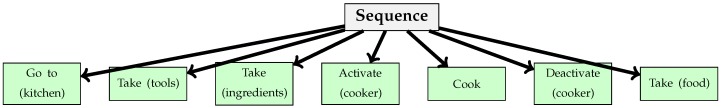
Behavior trees that manage a cooking scenario. In this Figure, the names between the parenthesis represent parameters. The node “cook” plays cooking animation.

**Figure 7:**
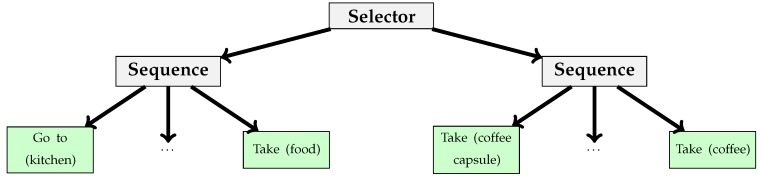
Extension of scenario in Figure 6. In this Figure, the names between the parentheses represent parameters.

According to these components we can build the example scenarios shown in Figure 6 and Figure 7. Figure 6 defines a simple cooking scenario. In this one, the inhabitant will try to cook the requested food. He/she cannot because an ingredient is missing, for example. He/she will finish the activity. Figure 7 presents an extension of the previous scenario where the inhabitant will try to cook and if he/she cannot, he/she tries to make coffee.

We use the same principle to define devices’ behavior. For electric devices, we consider the following behavior components:

- **Increase electric consumption**: The device increases its electric consumption.
- **Stay idle**: The device does not change its electric consumption for a given period of time.
- **Decrease electric consumption**: The device decreases its electric consumption.

**Figure 8:**
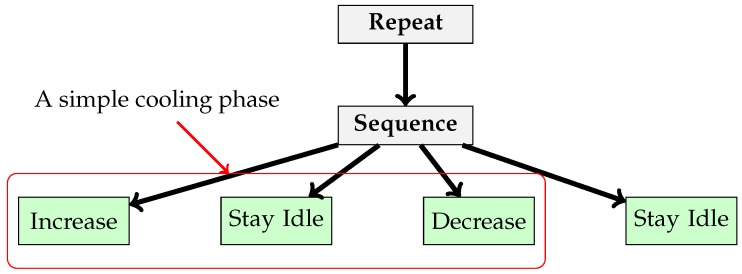
Behavior trees that manage refrigerator behavior.

With these components, we can define the behavior of a refrigerator given in Figure 8. We use this behavior in the scenario introduced in Section 5.

In order to define occupants and devices behaviors, we provide users with a set of behavior components. Users can use these components and define a scenario by using composites to order events. Table 4 presents a subset of these components.

Behavior trees are very interesting in this context because each behavior component can be configured to adjust the event order, for example, the time of completion, items used, etc. Moreover, users just have to add a new composite logic to create specific occupant behavior. For example, the sequence logic imposes success on previous components to entities in order to start the next behavior of the sequence. That means that when the entity finishes the last component, all previous components have been done with success. Users can create a version of this logic where another behavior can be started, despite the fact that a component ends with failure. This logic will simulate the behavior of an occupant who does not correctly complete his/her tasks.

**Figure 9:**
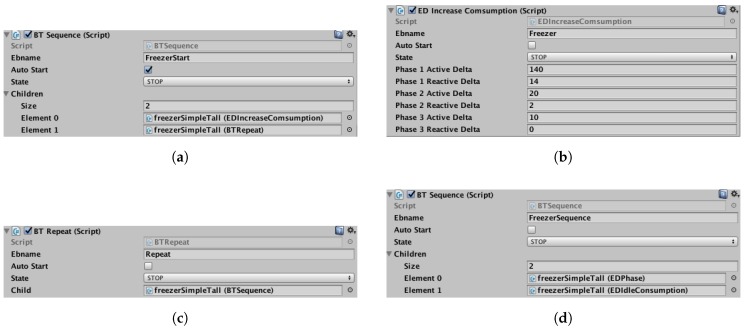
Screenshots of behavior tree edition in the Unity inspector. (**a**) Composite node; this element is the root of this tree; (**b**) The first son of the node in Figure 9a; this element increases the electricity consumption; (**c**) The second son of the node in Figure 9a and composite node “*repeat*”; (**d**) Son of node in Figure 9c; this composite node manages the cooling phase of our refrigerator and creates its electric signature.

Our solution provides users with two ways to configure behavior trees. The first one is by scripting; the user writes a script where he/she creates the behavior tree. The second way is by using the Unity inspector. We provide a collection of scripts that have to be linked to an entity with this inspector. Figure 9 shows screenshot of this script in the inspector.

### 4.4. External Elements

Our simulator allows external programs to interact with the virtual environment. This feature can be useful for defining experiments involving a smart home AI, for example. This AI will read the data gathered by sensors and act on actuators in response to particular situations. In order to perform this behavior, our simulator provides users with an actuator database. In this database, each actuator ID and the action to apply are stored (this action can be null if nothing has to be done). The external program has to add the action to do in order to act on actuators. Basically, our simulator reads this database to collect actions to perform.

### 4.5. Smart Home Design

**Figure 10:**
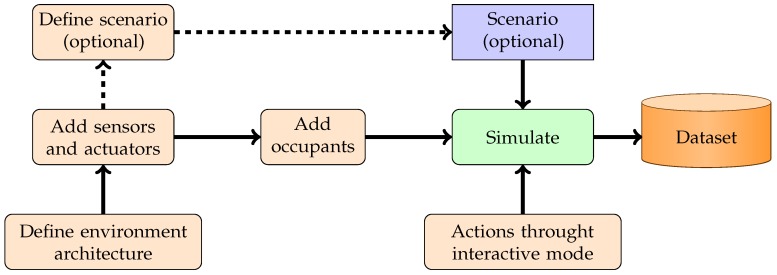
Scheme giving an overview of the design process.

Figure 10 resumes the main steps to design a virtual intelligent environment with our solution. The process is the same as the design for a real smart home. First, the designers define the architecture of the environment. Then, he/she selects and adds sensors and actuators to this environment. The users can complete these steps within the Unity interface by using our prefabs. He/she can also use our editor. The next steps consist of selecting the number and profiles of occupants. In parallel, users can use behavior trees to define his/her scenarios. These steps require the Unity editor. When the user launches simulation, he/she can control an occupant and/or let simulations play behavior trees to generate datasets.

### 4.6. Multi-Occupant Support

Our simulation supports multiple occupants in two ways:

- Model-based: In this approach, the user can give different behaviors to the occupants. These behaviors can make them collaborate in multi-person activities or perform solo activities.
- Hybrid: In this approach, the user can manually control an avatar (with his/her keyboard and mouse) and evolves in the environment with occupants controlled by scripts.

In both approaches, occupants evolve at the same time in the same environment. The simulator generates datasets that contain events that are created by different characters.

### 4.7. Implementation

We use the Unity game engine to build our simulator. This project is open source and available for download at https://github.com/Iannyck/shima. Our project uses a SQLite database to store the dataset and another database to allow external programs to communicate with our virtual IE. Basically, each actuator has a set of states that is stored into a database. A manager reads the state in the database and then changes the state of the actuator in the virtual IE.

**Figure 11:**
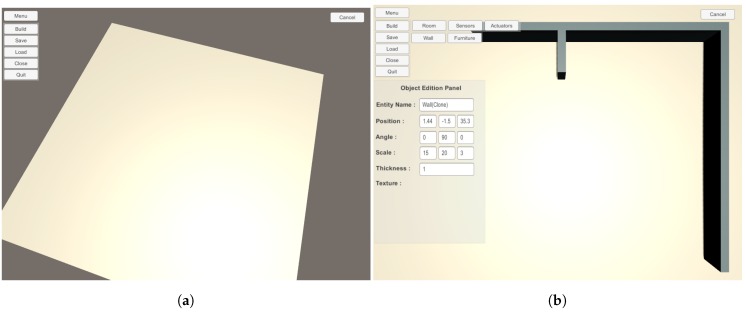
Screenshots of our editor for designing an intelligent environment. (**a**) Empty environment; (**b**) Environment with walls.

**Figure 12:**
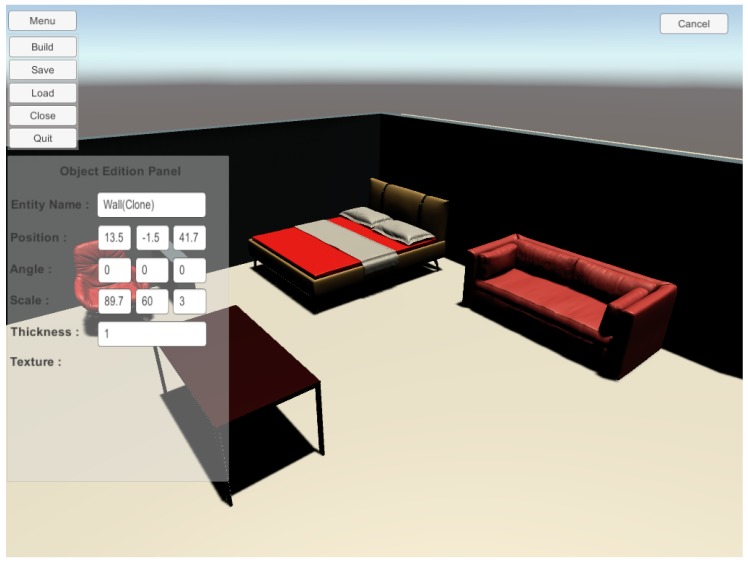
Screenshots of our editor for designing an intelligent environment with walls and furniture.

We have implemented two ways to design intelligent environment with our solution. The first one consists of using the Unity 3D game engine and using our prefabs to create a new scene. The advantage of this solution is the possibility of adding new elements easily such as particular furniture or textures. The second method consists of using our 3D editor. With our editor, the user is not required to install unity 3D. Our editor is a light version of the Unity 3D editor. It is focused on options to add and configure locations and sizes of items. It does not provide elements to change visual aspect of items for example. It is also possible to start a scene with our editor and finish it with the Unity 3D editor. The opposite scenario is possible if the user uses only our prefabs or adds his/her own to the editor database. Figure 11 and Figure 12 show screenshots of our editor.

## 5. Example

In this section, we propose a scenario example to show the data generated. Firstly, we will describe the architecture and sensors’ location in the next subsection. Then, we introduce our simulation scenario in Section 5.2 and we present generated data in Section 5.3.

### 5.1. Settings

**Figure 13:**
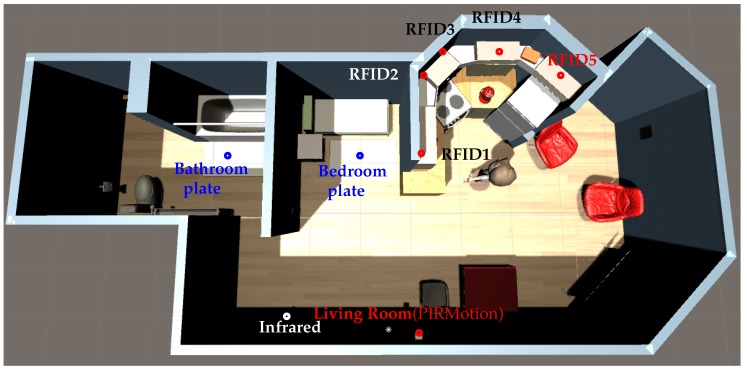
Screenshot of the home architecture for our scenario and the location of sensors.

**Figure 14:**
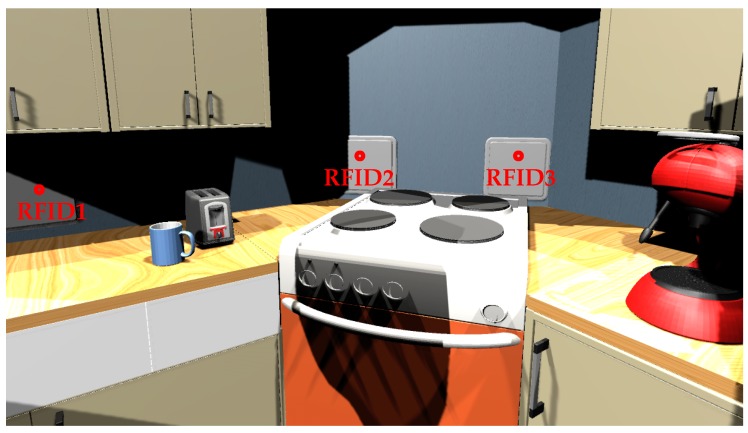
Screenshot of RFID antennas, front view.

**Figure 15:**
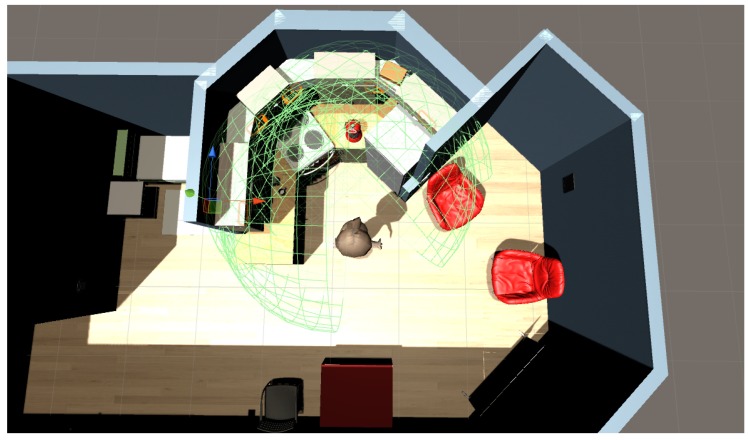
Screenshot of RFID antenna range.

We use a small flat as the place for this scenario. Figure 13 shows a picture of the home architecture and sensors locations. Table 5 presents the list of sensors. We use two pressure plates in the bedroom and bathroom, a flow meter in the flush toilet and for the bathroom faucet, contact sensors on shelves, one infrared sensor, one PIR motion, and five RFID antennas. This RFID antenna uses a token ring protocol. In this token ring, each antenna is activated at 0.2 s and the noise_function() alters the RSSI with a value in the interval [0, 3]. Figure 14 shows another view of RFID antennas and Figure 15 shows an RFID antenna range. We can note that the refrigerator is an obstacle in front of RFID antenna number 5. We set a naive degradation by this obstacle; it degrades signal strength by −20.

Table 6 presents our electric devices with their configuration. The sampling frequency is four reads per second.

### 5.2. Scenarios

We perform a simple scenario that involves cooking, use of the flush toilet and bathroom sink, eating, and sleeping. The avatar starts in the kitchen and visits the flat for a few seconds. The next steps are as follows:

- He goes to the bathroom and uses the flush toilet and then the bathroom sink to wash his hands.
- He starts a cooking activity. In this activity, he uses the coffee machine, takes tools, opens refrigerator to take ingredients, and uses the toaster and the cooker. During cooking, he drinks something in his mug.
- Once the food is ready, he takes a dish, a mug, a spoon and goes to the living room. During dining, the avatar at times goes to the refrigerator to get water.
- He tidies the table, then goes to the bathroom, and then goes to bed.

### 5.3. Result

**Figure 16:**
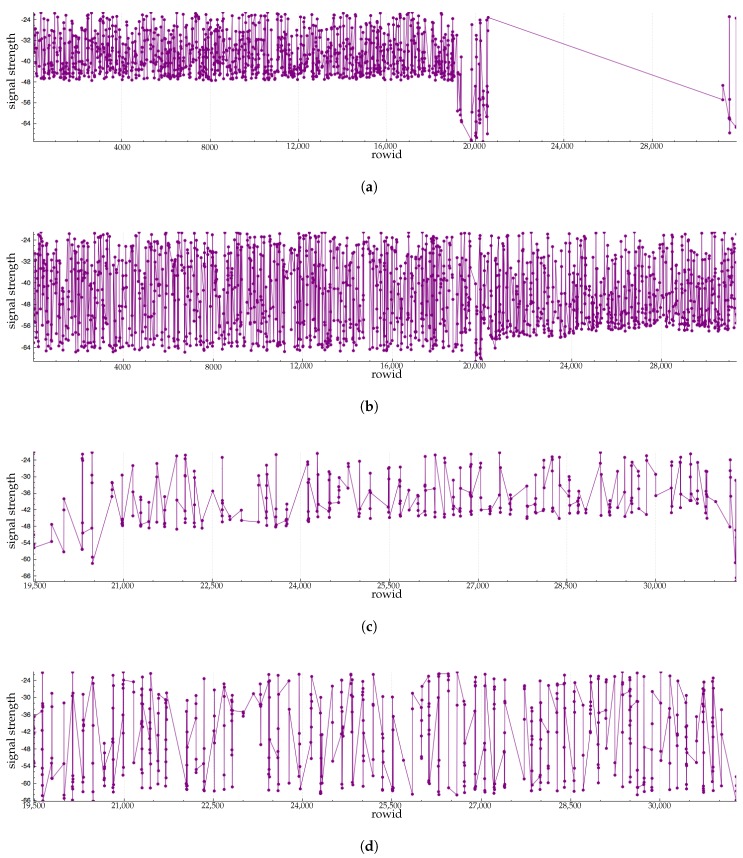
Sample of charts of RFID antenna signal strength for the tag named “TagMug”. This is the raw data of signal strength. (**a**) Antenna RFID1; (**b**) Antenna RFID2; (**c**) Antenna RFID3; (**d**) Antenna RFID4.

**Figure 17:**
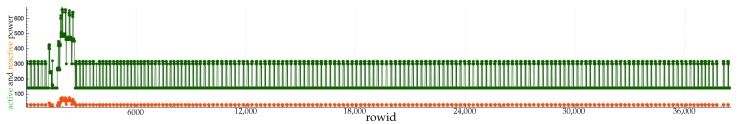
Evolution of electricity consumption in phase 1. Figure 20 details the variation around the spike.

**Figure 18:**
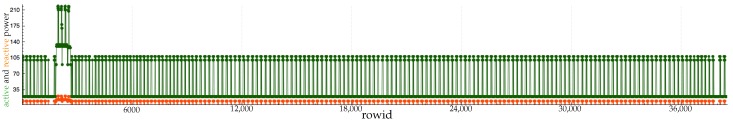
Evolution of electricity consumption in phase 2.

**Figure 19:**
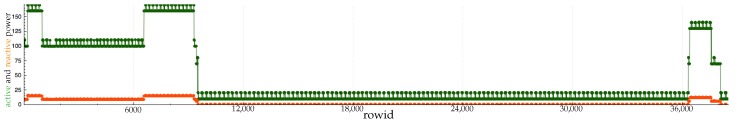
Evolution of electricity consumption in phase 3.

**Figure 20:**
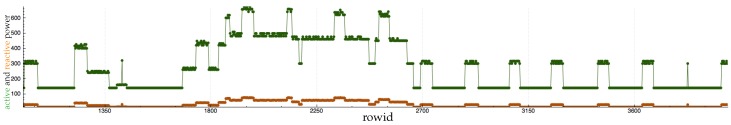
Evolution of electricity consumption in phase 1 during the cooking activity.

Figure 16, Figure 17, Figure 18, Figure 19 and Figure 20, and Table 7 and Table 8 show datasets generated by these scenarios.

Antenna RFID1 and RFID2 have recorded more data for this tag than antennas RFID3 and RFID4. This is due to the fact that these antennas can detect mugs at the beginning of the scenario. On RFID1 charts, the gap after the mid of the chart corresponds to the time when the avatar goes to the living room with the mug. Tracks of antenna RFID3 and RFID4 correspond mainly to the moves of the mug during the cooking scenario.

Figure 17, Figure 18 and Figure 19 show the evolution of electricity consumption during the whole scenario. In Figure 17, we can note that after a spike the pattern remains the same for the rest of the scenario. This is because the refrigerator is the only device in this phase which requires electricity. In phase 3 (Figure 19), the consumption around of 25 watts corresponds to the time when the occupant is sleeping. All lights are off, and the refrigerator is the only electric device with lamps that acts in phase 3 in our scenario.

In Figure 20, variations in consumption correspond to activation of the coffee machine, refrigerator (due to the door being opened and closed), the toaster, and the cooker. The cooker is the last device deactivated.

Infrared activation allows us to detect when the occupants go to the bathroom and leave it. Table 7 shows data recorded by this sensor.

Table 8 shows activation of binary sensors. The PIR motion sensor is activated mainly when the occupants visit the place, go to the bathroom, cook, and eat.

## 6. Application and Discussion

We can use this simulator in different kinds of studies in intelligent environment domain. In the activity of daily living (ADL) recognition field, this simulator can be used to develop a new approach for recognition. Indeed, in this domain, researchers aim to propose solutions to deduce occupants’ activities. ADL recognition methods try to detect activities patterns in data gathered from sensors [38,39]. These solutions have to be accurate, reliable, robust, and not require expensive sensors to be widely implemented.

In order to design these approaches, designers have to study how activities affect sensors and discover activity patterns. However, activity recognition methods are very specific. That means these approaches are designed for a specific set of activities, for a particular set of sensors, and for particular user profile. Consequently, researchers need to use datasets generated in similar conditions (i.e., with the same type of sensors, and the same user profile performing target activities). Access to the right database with sensors in particular configurations or for a particular profile can be very difficult in some cases. Consequently, researchers can use this simulator to generate their dataset. They are able to set the architecture of the place and location of sensors, and define the occupant’s behavior to simulate the right user profile. Moreover, researchers can act on sensor configurations to alter the noise in the dataset in order to evaluation robustness and reliability of their approach.

It is important to keep in mind the fact that values generated by virtual sensors are not equal to those generated by real sensors. That means if we put a tag at around 1 m in front of an RFID antenna, a real sensor can generate an RSSI in the range [–20, –30] according to interference in the environment, whereas our virtual sensors will provide a value equal to –20.7599 without noise, for example.

**Figure 21:**
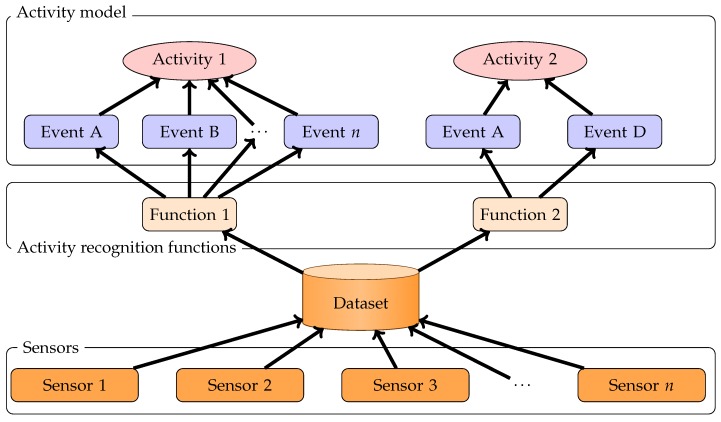
Split of activity in event and module that detects events through sensor data.

One approach in ADL recognition is to define an activity as a set of events. For example, a cooking activity involves a cooker, and the movement of kitchen tools such as knives, pans, spoons. Making coffee implies a coffee machine and a mug. The second step is to identify these events by analyzing data generated by sensors. Basically, event recognition can be seen as a function that takes a vector of sensor data as an argument and it returns events that are recognized. Figure 21 resumes this approach.

By using the simulator, we can for example evaluate a naive function that detects cooking activity. This function detects cooking activity according to: (1) the cooker electric consumption pattern; and (2) items with an RFID tag that are in the group of kitchen tools that moves or is near the cooker. In order to recognize cooker electric consumption we use a naive approach that monitors electricity evolution on each phase. Each time a significant change occurs, our function compares if this change matches with a cooker phase. This function returns “the cooker is on” if it detects cooker phases. In order to detect movement of items and their proximity with the cooker, we filter the RFID RSSI with a moving average. We use triangulation to compute location of items. If they are in a defined area, we consider they are near the cooker. If there is a signification change in location, we consider that they are moving.

We use a simple scenario to generate a dataset to run this approach. This scenario is performed in the environment introduced in the previous section. The main steps of this scenario as follows:

1. The occupant cooks. He/she uses the cooker and a pan with a spoon.
2. The occupants turn off the cooker, then he/she does not move for a few seconds.
3. The occupants turn on the cooker, then he/she does not move for a few seconds.
4. The occupant turns off the cooker, then he/she cooks. He/she uses the cooker and a pan with a spoon.

**Figure 22:**
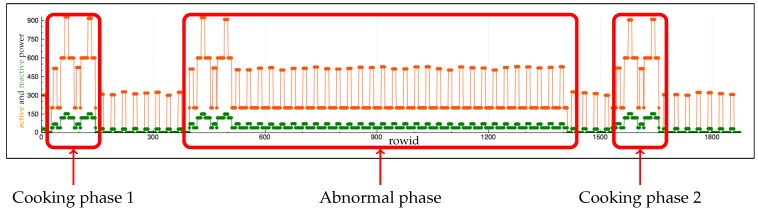
Sample of electricity data.

**Figure 23:**
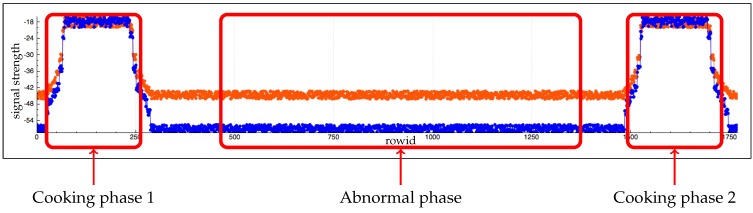
Sample of the received signal strength indication (RSSI) of antennas RFID2 and RFID3.

Step 3 introduces an abnormal event. Our recognition function must not return a cooking activity for this step. Figure 22 and Figure 23 show samples of data generated by this scenario. Table 9 shows results of our algorithm.

The simulator can be used to generate scenario and evaluation accuracy and recall of a function. With simulators, it is possible to experiment several times by adding some variations such as noise or order of activities. For example, we can use the same scenario but increase the noise in RFID and electricity to evaluate the robustness of a proposition.

In the assistance field, researchers can use this simulator to study the behavior of the intelligent environment they are designing. In this case, researchers have to design the architecture of the environment, set sensors and actuators, and design AI. In parallel ways, they have to design avatars’ behaviors. Basically, the simulation of interaction between occupants and environments will allow researchers to perceive reaction of the system according to each situation and to evaluate their AI.

For example, if we take the previous scenario with cooking activity, when the system detects the event cooker is on but there is no cooking activity, researchers can study impact of several propositions according to inhabitants’ behaviors.

Moreover, simulation tools can be used in combination with physical devices such as smart devices. In a simple way, these devices can provide users with a new input medium. For example, smart speakers can be used to control avatars or virtual devices by voices. That requires to implement an interface that maps voice commands to actions.

In a more advanced approach, physical and virtual devices can be combined to build hybrid laboratories. Hybrid laboratories propose some advantages to researchers. On the one hand, researchers can complete their installation with virtual devices to experiment more scenarios. For example, it is possible to use the simulator to add virtual rooms to an existing building. Obviously, these virtual rooms will be used only by virtual entities. However, that allows researchers to run scenarios where AI analyzes data gathered from virtual sensors and act on physical devices

On the other hand, they can quickly change the configuration of the laboratory. Indeed, as a part of the configuration is virtual. Consequently, researchers can save different configurations in different files and switch between these files according to current needs.

It is also possible to run multi-occupant scenario with real and virtual occupants. These kinds of scenarios can be used to study user experience in particular situations.

## 7. Conclusions and Future Works

In this paper, we presented our IE simulator. This simulator allows IE researchers to generate datasets in order to evaluate their contributions. Moreover, it allows IE designers to evaluate their IE proposition before the implementation of the new one. Our simulator allows the building of the environment (position of walls, doors, windows and selection of items) and the selection and positioning of sensors. It proposes selection with several kinds of sensors: RFID, ultrasound and infrared sensors, pressure plates, contact sensors, flow meters, and movement detectors. These sensors generate data similar to those generated by physical sensors.

Our simulator proposes two modes to generate datasets. The first one is an interactive mode where the user controls an inhabitant and can interact with items in the home. The second mode is model-based, whereby the inhabitants are controlled by scripts. We use behavior trees to implement these scripts. With this method, it is easy to create complex behaviors because these behaviors are a structured aggregation of simpler components. It allows reuse of components to build new ones. Moreover, the components can have several parameters that allow users to create variation among the same scenario. Our simulator is open source and available at https://github.com/Iannyck/shima.

This paper includes a scenario in order to show an example of datasets generated. We use this scenario to generate a dataset. It consists of activities such as cooking, use of the faucet, and eating. These scenarios imply several sensors that are set in the kitchen, the bathroom, and the living room.

For future works, we aim to improve our multi-occupant support by allowing users to control several avatars. That will help to decrease the designing time because it will not be mandatory to use a model-based approach to define the scenario. Human users will be able to collaborate and adapt the scenario during the simulation. We also want to provide users with the possibility of using the interactive approach to define the scenario. In fact, users will control avatars and the sequence of actions will be recorded in order to allow users to replay the sequence.

We want to add a fast-forward mechanism. A fast-forward mechanism can help users to generate datasets that represent a large period of time (for example several weeks) in a short time. With a fast-forward mechanism and environment event, it will be possible to simulate environment evolution in scenarios.

Finally, we aim to add virtual reality support in order to increase immersion during simulations through an interactive mode. Virtual reality will help users to feel as if they are in a real smart environment, and can make some actions seem easier and more natural [40].

## Figures and Tables

**Table 1:** Sumary of propositions according to our criteria. Act script: actuator scripting; Env script: environment scripting; Eng ext: engine extension; NA: not applicable.

Tool	Approach	Sensors	Multi-Occupants	Env Script	Act Script
OpenSHS [8]	Hybrid	Binary	Partial	Eng ext	Eng ext
Park et al. [9]	Interactive	NA	no	no	no
IE Sim [10,11]	Hybrid Interactive	Binary	no	no	no
Kormanyos et al. [12]	Model-based	Binary, numeric (water, electricity, RFID, temperature), internal variable (water need, etc.)	no	no	no
PerSim 3D [13]	Model-based	Binary	no	no	no
UbikSim [21,22]	Model-based	Binary	yes	Eng ext	Eng ext
Ariani et al. [14]	Interactive	Binary	yes	no	no
SIMACT [15]	Model-based	Binary	no	Eng ext	Eng ext
Our proposition	Hybrid	Binary, numeric	yes	yes	yes

**Table 2:** List of sensors provided by our simulator. PIR: passive infrared.

Type	Value	Description	Simulation Model
RFID	Numeric	Strength of the signal between the antenna and the RFID tag	Collision box + raycast
Pressure plate	Boolean	*true* if an entity pressures	Collision box
PIR motion		*true* if an entity moves in its range	Collision box + Raycast
Contact		*true* if the two parts are connected	Event listener
Flow meter		*true* if an faucet is opened	Event listener
Electricity	Numeric	Active and reactive power on a line	Environment variable
Infrared	Numeric	Distance where the beam is stopped	Raycast
ultrasound			

**Table 3:** Sample of behavior components.

Composite Logic	Succeeded	Failed	Running
Sequence	All children succeeded	At least one child failed	At least one child running
Selector	At least one child succeeded	All children failed	At least one child running and others failed
Repeat	Enough repeats succeeded	Not enough repeats succeeded	Child is running
Inverter	Child failed	Child succeeded	Child is running

**Table 4:** Sample of behaviour components.

Component Name	Description	Parameters
Cook	Uses tools and ingredients to cook a dish	Time
GoTo	Goes to a given place	Place to go, speed
Activate	Activates a device	Device to activate
Take	Takes an object	Object to take
Clean	Cleans an object	Object to clean, time
Increase consumption	Increases electric consumption	Delta value
Decrease consumption	Decreases electric consumption	Delta value
Stay idle	Does not change electric consumption	time

**Table 5:** List of sensors in our example. Figure 13 shows position of sensors.

Room	Sensor	ID
Kitchen	RFID	RFID1
	RFID	RFID2
	RFID	RFID3
	RFID	RFID4
	RFID	RFID5
	Contact sensor	Kitchen shelf top left
	Contact sensor	Kitchen shelf top middle
Bedroom	Pressure plate	Bedroom plate
Living room	PIR motion	Living room
	Infrared	Infrared
Bathroom	Flow meter	Bathroom sink
	Flow meter	Flush toilet
	Pressure plate	Bathroom plate

**Table 6:** List of electric devices with their configuration in our example.

Devices	Phases Used
Electric cooker	1,2
Refrigerator	1,2,3
Coffee machine	1
Toaster	1
(4) Lamps	3

**Table 7:** Sample of the infrared dataset generated by our example.

Timestamp	Distance
05:57:00:134	4.469549
05:58:49:318	9.904987
05:58:49:772	9.194394
06:10:19:295	10.56265
06:10:26:299	6.078778
06:10:30:196	11.6572
06:10:30:613	9.915684
06:53:32:471	4.650184
06:55:36:141	8.025949

**Table 8:** Sample of the binary sensors dataset generated by our example. The first column is the timestamp, the second column provides the sensor name, the third shows the type of sensor, and the last column shows the state of the sensor at the current timestamp.

Timestamp	SensorId	Type	Value
05:56:45:625	Living room	PIRMotion	true
05:56:48:341	Living room	PIRMotion	true
05:56:53:743	Living room	PIRMotion	true
05:56:54:818	Living room	PIRMotion	true
05:56:55:886	Living room	PIRMotion	true
…			
05:57:22:865	Bathroom plate	PIRMotion	true
05:57:24:132	Bathroom plate	PIRMotion	false
…			
05:58:15:98	Flush toilet	BinaryFlowMeter	true
05:58:16:124	Flush toilet	BinaryFlowMeter	false
05:58:21:866	Bathroom sink	BinaryFlowMeter	true
05:58:36:848	Bathroom sink	BinaryFlowMeter	false
05:58:41:57	Bathroom plate	PIRMotion	true
05:58:42:381	Bathroom plate	PIRMotion	false
05:58:48:138	Living room	PIRMotion	true
05:58:49:318	Living room	PIRMotion	true
05:58:50:458	Living room	PIRMotion	true
…			
06:00:09:836	Kitchen shelf top left	ContactSensor	false
06:00:15:855	Living room	PIRMotion	true
06:00:17:18	Living room	PIRMotion	true
06:00:31:252	Kitchen shelf top left	ContactSensor	true
06:00:47:218	Kitchen shelf top middle	ContactSensor	false
06:00:48:919	Living room	PIRMotion	true
…			
06:09:34:810	Bathroom plate	PIRMotion	true
06:09:36:393	Bathroom plate	PIRMotion	false
…			

**Table 9:** Result of our algorithm on data from Figure 22 and Figure 23.

Phase Name	Electricity Function	RFID Function
Cooking phase 1	Cooker	cooking tools moved
Abnormal phase	Cooker	
Cooking phase 2	Cooker	cooking tools moved
